# How the COVID-19 pandemic affects the moral reasoning of pediatric residents and the general population

**DOI:** 10.1186/s12909-023-04265-6

**Published:** 2023-05-24

**Authors:** M. G. Jean-Tron, D. Ávila-Montiel, Titto C. Hill-de, H. Márquez-González, G. Chapa-Koloffon, A. V. Ávila-Hernández, M. A. Núñez-Benítez, O. Muñoz-Hernández, J. Garduño-Espinosa

**Affiliations:** 1 Hospital Infantil Federico Gómez, Mexico City, Mexico; 2 Mexican Social Security Institute, Mexico City, Mexico; 3 National Autonomous University of Mexico, Mexico City, Mexico; 4 Dirección de Investigación, Tercer piso Edificio Hemato-Oncología e Investigación, Hospital Infantil Federico Gómez, Calle Dr. Márquez 162, Col. Doctores, Cuauhtémoc, CP 06720 Mexico

**Keywords:** Moral reasoning, Kohlberg’s theory, Moral development, Judments, Morality

## Abstract

**Background:**

Kohlberg’s theory of moral development asserts that people progress through different stages of moral reasoning as their cognitive abilities and social interactions mature. Individuals at the lowest stage of moral reasoning (preconventional stage) judge moral issues based on self-interest, those with a medium stage (conventional stage) judge them based on compliance with rules and norms, and those at the highest stage (postconventional stage) judge moral issues based on universal principles and shared ideals. Upon attaining adulthood, it can be considered that there is stability in the stage of individuals’ moral development; however, the effect of a global population crisis such as the one experienced in March 2020, when the World Health Organization (WHO) declared the COVID-19 pandemic, is unknown. The purpose of this study was to evaluate the changes in the moral reasoning of pediatric residents before and after one year of the COVID-19 pandemic and compare them with a general population group.

**Methods:**

This is a naturalistic quasi-experimental study conducted with two groups, one comprised 47 pediatric residents of a tertiary hospital converted into a COVID hospital during the pandemic and another group comprised 47 beneficiaries of a family clinic who were not health workers. The defining issues test (DIT) was applied to the 94 participants during March 2020, before the pandemic initiated in Mexico, and later during March 2021. To assess intragroup changes, the McNemar-Bowker and Wilcoxon tests were used.

**Results:**

Pediatric residents showed higher baseline stages of moral reasoning: 53% in the postconventional group compared to the general population group (7%). In the preconventional group, 23% were residents and 64% belonged to the general population. In the second measurement, one year after the start of the pandemic, the group of residents had a significant decrease of 13 points in the P index, unlike the general population group in which a decrease of 3 points was observed. This decrease however, did not equalize baseline stages. Pediatric residents remained 10 points higher than the general population group. Moral reasoning stages were associated with age and educational stage.

**Conclusions:**

After a year of the COVID-19 pandemic, we found a decrease in the stage of moral reasoning development in pediatric residents of a hospital converted for the care of patients with COVID-19, while it remained stable in the general population group. Physicians showed higher stages of moral reasoning at baseline than the general population.

**Supplementary Information:**

The online version contains supplementary material available at 10.1186/s12909-023-04265-6.

## Introduction

As human beings, we are capable of making decisions about good and bad, right and wrong. This capacity is formed throughout life and constitutes the morality of people [1]. Specifically, moral reasoning is defined by Kohlberg as judgments about right and wrong [2], moral reasoning is important because it will allow people to act freely and responsibly in all aspects of their lives [3]. Moral reasoning is expected to be greater in some professionals, such as doctors because they must place the interest of the patient above their own to help the patient and contribute to the common good and society [4, 5]. Kohlberg’s theory of moral development states that people progress through different stages of moral reasoning as their cognitive abilities and social interactions mature, however, the rate of progression and the final stage reached varies from person to person [6]. Kohlberg’s contribution to moral psychology has been applied to Piaget’s stage development scheme for studying thinking about how moral judgment evolves in the individual. [7]

Individuals at the lowest stage of moral reasoning (preconventional stage) judge moral issues based on a self-interest scheme, those with a medium stage (conventional stage) judge them based on compliance with rules and norms, and those at the highest stage (postconventional stage) judge moral issues based on universal principles and shared ideals [2, 6, 8] **(**Table 1**)**.

Kohlberg’s theory, being validly explanatory of the structural-evolutionary pattern of morality, implies that, upon reaching and consolidating a stage, the individual will not return to a previous one; this characteristic was verified by Kohlberg himself in his longitudinal studies [9], later James Rest in his extensive studies recognized the presence of these stages, however, he mentioned that it would be better to refer to a predominant stage in terms of probability [10]. There have been multiple studies evaluating these stages of moral reasoning in both adolescents [11] and adults [12–16].

Kohlberg and Colby established that the conventional stage of moral reasoning is most prevalent in the adult population, whereas the preconventional stage is the most prevalent in children and young adolescents, and stage 6 is only found in a minority of the adult population. This stage can only be attained after the age of 20, after reaching a stage of formal operations (Piaget) and having received sufficient social stimuli [8, 17].

**Table 1:** Kohlberg’s stages of moral development

Preconventional stage	
Stage 1	Avoidance of punishment. The physical consequences of the act determine whether it is good or bad.
Stage 2	Instrumental exchange. Right actions are those that instrumentally satisfy one’s own needs. People are valued in terms of their usefulness.
**Conventional stage**	
Stage 3	Interpersonal conformity. Right actions are those expected by society or peers, with the purpose of obtaining the approval of others.
Stage 4	Law and order. Right actions consist of doing the right thing, respecting authority and maintaining social order. Deviation from the rules can lead to social chaos.
**Postconventional stage**	
Stage 5	Social contract. Behavior is guided by a sense of obligation to a social contract that protects people’s rights. Laws and obligations should be based on the rational calculation of global utility, “the greatest good for the greatest number”
Stage 6	Universal ethical principles. Right actions are defined in terms of universal moral principles (justice, fairness of human rights, and respect for the dignity of human beings as individuals).

In March 2020, the World Health Organization (WHO) declared a state of COVID-19 pandemic [18]. To coordinate and integrate the hospital response for the care of patients with COVID-19, it was announced that a hospital reconversion model would be carried out in Mexico on March 29 and that the “blinded” would initially be the National Institute of Health that would manage patients under 18 years of age with severe COVID-19 [19]. Therefore, the resident physicians of the said institution would have activities in the first line of care for patients with COVID-19.

Generally, resident physicians are exposed to factors that negatively affect their personal life, such as long working hours with up to 36-hour shifts, living far from their place of origin, insufficient support networks, work stress, and making difficult clinical decisions. This can lead to moral distress and anxiety, which can be exacerbated in a crisis such as the COVID-19 pandemic [20]. Mental health is often affected in epidemics due to reactions, such as fear of becoming sick and/or dying or that a loved one becomes sick or dies, uncertainty about the economic situation, boredom, loneliness, and depression due to isolation [21]. Moral reasoning depends on cognitive skills, including general reasoning, and social interactions [6]. It is known that reasoning can be affected by mental illness or by crises such as the COVID-19 pandemic [22, 23]. Specifically, the COVID-19 pandemic, especially in its first year, has been a challenge for the world in terms of moral decision-making in public health, such as deciding who should be treated first or who should receive a ventilator [24]. It is not only important that the great health authorities have a higher level of moral reasoning, but also those who care for the patient from the front line since they are the ones who will execute the decisions made by the authorities.

The purpose of this study was to evaluate changes in the moral reasoning of pediatric residents before and after one year of the COVID-19 pandemic and compare them with a general population group.

## Methodology

It is a prospective quasi-experimental naturalistic study, it was classified in this way because the naturalistic intervention (COVID-19 pandemic) occurred circumstantially and was not manipulated or under the control of the researchers [25]. Likewise, randomization was not carried out, unlike the experimental studies [26] since all the participants in this study were exposed to the natural intervention, and the measurements were made before and after said event. This type of design has been widely used in public health studies, however, it has also been used in other areas such as economic, social, and psychological, among others [27].

The study was made up of two groups, one made up of 47 pediatric residents of a third-level hospital converted into a COVID hospital during the pandemic and another group made up of 47 beneficiaries of a family clinic who were not health workers, who made up the group. “general population” group. The study was approved by the Ethics and Research Committees of the Hospital Infantil de México Federico Gómez and all participants were invited to participate voluntarily, explaining the study to them and obtaining informed consent. Specifically, it was emphasized to the group of pediatric residents that not participating or withdrawing from the study would not have implications for their academic activities.

### Participants

*Pediatric resident group.* For convenience, all students enrolled in the first year of the Pediatrics specialty in the “blinded” institution during the 2020–2021 school year were included. Two days before starting their first-year course, they were invited to participate in the study, clearly explaining the procedure and content of the questionnaires. Informed consent was obtained from the 49 registered residents; however, one was eliminated for not answering the follow-up questionnaire and another because the questionnaire was invalid due to inconsistencies in responses, leaving a total of 47 subjects.

*General population group.* The beneficiaries of a family clinic who attended a consultation between February and March 2020 were invited to participate, including subjects of legal age who did not have any disabilities that prevented them from answering the questionnaires. Subjects who accepted and met the criteria for selection were clearly explained what their participation consisted of and were asked to sign the informed consent letter. A total of 61 questionnaires were completed, of which 13 (21%) were annulled, leaving a total of 48. Of those annulled, 9 questionnaires were annulled due to incompletion, 3 due to inconsistencies, and 1 due to exceeding the allowed M-score. One participant was subsequently eliminated because they were unavailable for the second measurement, leaving a total of 47 subjects.

### Questionnaires

The questionnaires were applied by trained and qualified psychological personnel. They were conducted in hospital facilities for the pediatric resident group and in family clinics for the general population group. Two questionnaires were applied to all the participants. The first one was used to obtain general data that included age, sex, place of residence, religion, and monthly income.

The second questionnaire was used to measure the stage of moral reasoning. The defining issues test (DIT) was used in its short version (supplementary material), designed by James Rest based on Lawrence Kohlberg’s theory [10]. In this questionnaire, the subject is presented with three stories, each one facing a moral dilemma. In the first section of answers, subjects were asked their opinion on what the person in the story should do, being able to answer “yes,” “no” or “I cannot decide.” In the second section, subjects were asked to give their opinion according to the degree of importance of twelve statements, which represent each of Kohlberg’s moral stages. These statements pose possible resolutions to the dilemma. In the third section, subjects chose the four most important statements in each story based on what was chosen in the second section and ranked them from first to fourth in decreasing order of importance. With these answers, raw and percentage scores were elaborated that express the frequency with which the subject used one stage or other stages 2 to 6 of moral reasoning.

The principled moral reasoning index (P index) was also calculated, which expresses the degree to which a person judges these problems from the postconventional perspective. This index was developed with scores corresponding to stages 5 and 6. Likewise, categorization of the P index was conducted in the three stages of development of moral reasoning. The preconventional stage had scores lower than 30 points, the conventional stage had scores between 30 and 40 points, and the postconventional stage had scores greater than 40 points. The M score was calculated, which is representative of meaningless items, this score does not represent any stage, but rather the subject’s tendency to approve statements for their claims, rather than for their meaning, a high M score serves to recognize that the subject is not taking the quiz very seriously, so a raw M score greater than 4 invalidates the test.

The DIT has been validated in Mexico and other Latin American countries. Results show an adequate internal consistency, with a Cronbach’s alpha of 0.71, as well as the test-retest procedures of 0.65 [11, 28], which are similar to those obtained by Rest with the DIT in its original version: a Cronbach’s alpha coefficient of approximately 0.70 and test-retest reliability between 0.70 and 0.80 [29].

In the present study, two DIT measurements were made; the initial one from February to March 2020 prior to the COVID-19 pandemic in Mexico and the second one a year later from February to April 2021.

### Statistical analysis

A descriptive analysis was prepared for the sociodemographic characteristics of the population. Qualitative variables were reported as total numbers and percentages, and quantitative variables as mean and standard deviation. Moral development profiles based on medians and minimum-maximum ranges were elaborated because the variables did not comply with normal distribution. These medians were calculated for stages 2 to 6, as well as for the P Index for each group.

To evaluate differences between groups in the first and last measurements, Fisher’s exact test was used for qualitative variables and Mann-Whitney U for quantitative variables. McNemar’s test was used to evaluate intra-group changes before and after the first year of the pandemic. The McNemar-Bowker test was used for the differences between the stages of moral reasoning and the Wilcoxon test for the P index; a subanalysis of the latter was performed by the stage of moral reasoning, sex, and age. The statistical analysis was performed with SPSS v. 24. The stage of statistical significance was established with a p < 0.05.

## Results

### Baseline results

A total of 94 participants who had completed the two evaluations and whose questionnaires were valid were included: 47 for each group. There were no significant differences for sex between groups, the female sex was predominant with 70%. The rest of the sociodemographic variables did show a difference between the groups, with the average age of residents and the general population being 26 years and 45 years, respectively. The majority of residents cohabited with a roommate (53%) while the majority of the general population cohabited with a partner and children (74%). Most of the general population reported whether they professed, even moderately, their religion (98%), while only 57% of residents did **(**Table 2**)**.

**Table 2:** Sociodemographic characteristics of the study population

	Pediatric resident n = 47 TN (%)	General population n = 47 TN (%)
Sex		
Female	33 (70.2)	33 (70.2)
Male	14 (29.8)	14 (29.8)
Age (Mean, SD)	26 (1.7)	45 (17.1)
Marital status		
Single	44 (93.6)	10 (21.3)
CL marriage/Married	2 (4.3)	30 (63.8)
Divorced/ Separated	1 (2.1)	4 (8.5)
Widow(er)	0	3 (6.4)
Education		
WS/Elementary School	0	16 (34)
Middle school	0	16 (34)
High school/T	0	15 (31.9)
Bachelor’s degree	47 (100)	0
Occupation		
House work	0	16 (34)
Employee	0	24 (51.1)
Retired	0	2 (4.3)
Student	47 (100)	5 (10.6)
Income		
0–2699	0	5 (10.6)
2700–6799	0	30 (63.8)
6800–11,599	0	11 (23.4)
11,600–34,999	47 (100)	1 (2.1)
Religion	27 (57.4)	46 (97.9)
Cohabitants		
Alone	8 (17)	5 (10.6)
Couple and sons	0	35 (74.5)
Sons	0	4 (8.5)
Birth family	14 (29.8)	3 (6.4)
Roommate	25 (53.2)	0
Place of Birth		
Mexico City	18 (38.3)	36 (76.6)
Estado de México	5 (10.6)	3 (6.4)
Other state	20 (42.6)	0
Other country	4 (8.5)	0

TN: total numbers, CL: Common-Law, WS: Without studies, T: technician.

The baseline results of the DIT are shown in Tables 3 and 4. A significant difference was found in the P index, being 43.3 for pediatric residents compared to 23.3 for the general population. In both groups, it was observed that the score increases in the first stages until a higher score is obtained for stage 4, with a median of 36.6 for residents and a median of 40 for the general population. It is noteworthy that there are higher scores at the postconventional stage (stages 5a, 5b, and 6) for residents compared to the general population.

**Table 3:** Comparison Between Stage Groups and Baseline P Index

	Pediatric resident n = 47 median (min-max range)	General population n = 47 median (min-max range)	*p*
Stage 2	3.3 (0–23.3)	3.3 (0–20)	0.392
Stage 3	10 (0–53.3)	16.6 (0–43.3)	**0.001**
Stage 4	36.6 (0–63.3)	40 (23.3–70)	**0.023**
Stage 5a	23.3 (0–63.3)	13.3 (0–36.6)	**< 0.001**
Stage 5b	10 (0–13.3)	0 (0–13.3)	**< 0.001**
Stage 6	10 (0–20)	6.6 (0–20)	0.081
P Index	43.3 (0–76.6)	23.3 (3.3–50)	**< 0.001**

*Mann-Whitney U

Regarding the stages of moral reasoning, a significant difference was found between groups due to a higher percentage observed in the postconventional stage for residents (53%) than for the general population (only 6%), and a higher percentage observed in the preconventional stage (64%) for the general population (64%) than for residents (23%) **(**Table 4**)**.

**Table 4:** Comparison between groups in terms of baseline moral reasoning stages

Stage	Pediatric residents n = 47 TN (%)	General population n = 47 TN (%)	*p*
preconventional	11 (23.4)	30 (63.8)	**< 0.001**
conventional	11 (23.4)	14 (29.8)	
postconventional	25 (53.2)	3 (6.4)	

*Fisher’s exact; TN: total numbers.

### Results after the first year of the COVID-19 pandemic

When comparing the stages and the score after the first year of the pandemic, we found that the differences between the two groups persisted: the same profile was continuously observed in both groups, that is, with gradual increases from the first stages until reaching stage 4 and then decreasing. It is noteworthy that the scores obtained in the stages within the post-conventional stage (stages 5a, 5b, and 6) did not show as much difference as in baseline, except for stage 5b, which showed a significant difference. P index showed significant differences, having a median of 30 for pediatric residents compared to a median of 20 in the general population group **(**Table 5**)**.

**Table 5:** Final Comparison Between Stage Groups and P Index

	Pediatric residents n = 47 median (range min-max)	General population n = 47 median (range min-max)	*p* *
Stage 2	0 (0–13.3)	6.6 (0–26.6)	**0.002**
Stage 3	13.3 (0–46.6)	20 (0–46.6)	**0.006**
Stage 4	40 (0–70)	33.3 (16.6–63.6)	0.221
Stage 5ª	20 (0–43.3)	13.3 (0–40)	0.076
Stage 5b	6.6 (0–13.3)	0 (0–13.3)	**0.006**
Stage 6	6.6 (0–30)	6.6 (0–26.6)	0.826
P-Index	30 (10–63.3)	20 (6.7– 63.3)	**0.010**

*Mann–Whitney U

The results by moral reasoning stages can be seen in Table 6. Significant differences were found due to a higher percentage of pediatric residents in the postconventional stage (19%) compared to the general population (8.5%). 68% of the general population was found to be in the preconventional stage, whereas there were 42% of residents in this category.

**Table 6:** Final comparison between groups of stages of moral reasoning

Stage	Pediatric resident n = 47 TN (%)	General population n = 47 TN (%)	*p*
preconventional	20 (42.6)	32 (68.1)	**0.041**
conventional	18 (38.3)	11 (23.4)	
Postconventional	9 (19.1)	4 (8.5)	

*Fisher’s exact; TN: total numbers

### Intragroup changes before and after the first year of the COVID-19 pandemic

**Fig. 1:**
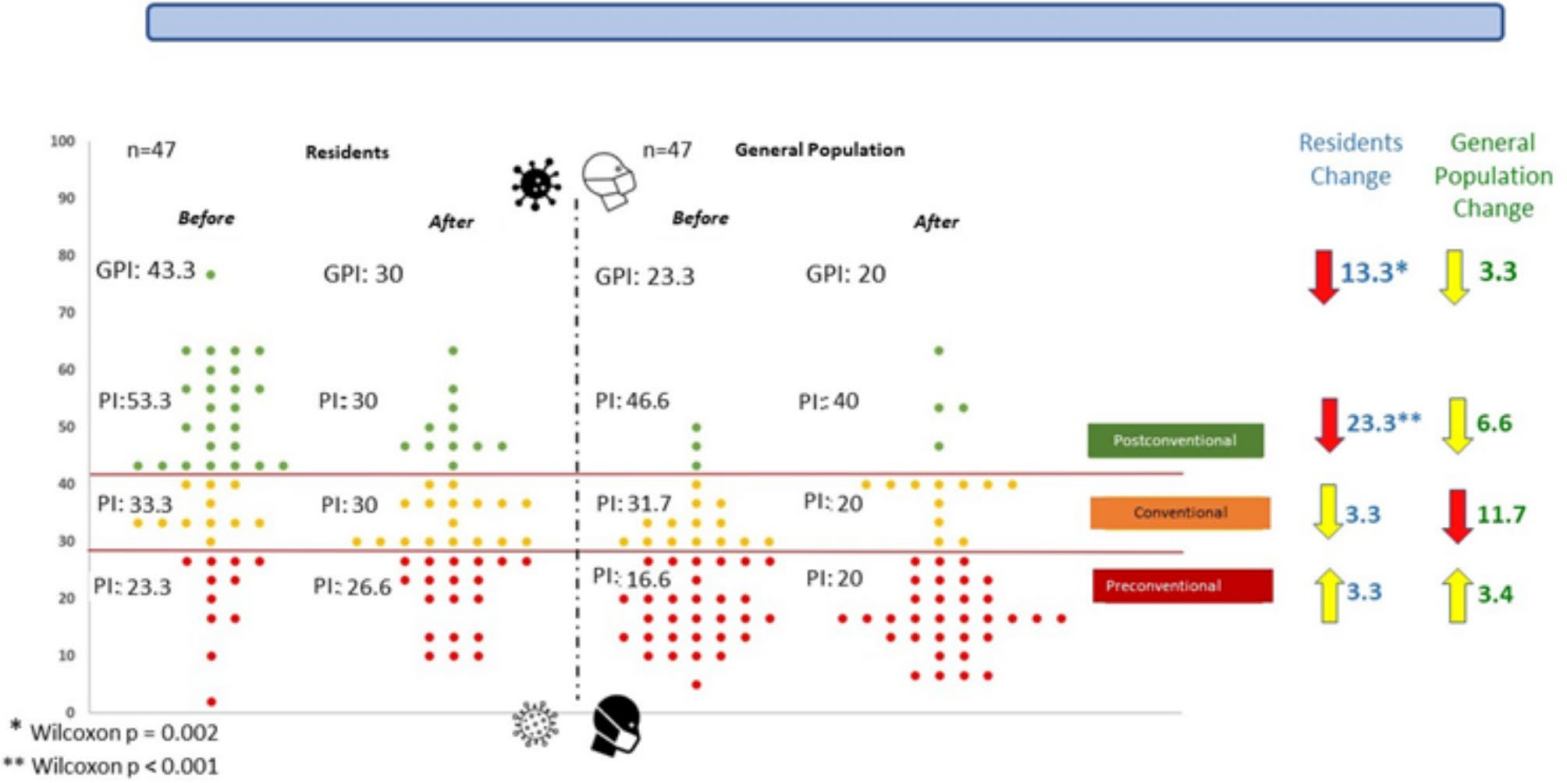
Differences in moral reasoning between pediatric residents and the general population before and after the 1^ST^ year of the COVID-19 pandemic

The changes in the medians of the P index in the group of pediatric residents before and after the first year of the pandemic are shown in Fig. 1. We found a P index decrease of 13 points, which was statistically significant (Wilcoxon test p = 0.002). Meanwhile, in the general population group, a decrease of 3 points in the P index was found without significant differences.

When evaluating the changes of the P index by stage of moral reasoning, we found a decrease of 23 points after the first year of the pandemic in the residents with a baseline postconventional stage (Wilcoxon test p < 0.001). In this same subgroup, no changes were observed within the general population. An 11-point decrease in the conventional subgroup was found in the general population; however, this was not statistically significant.

**Fig. 2:**
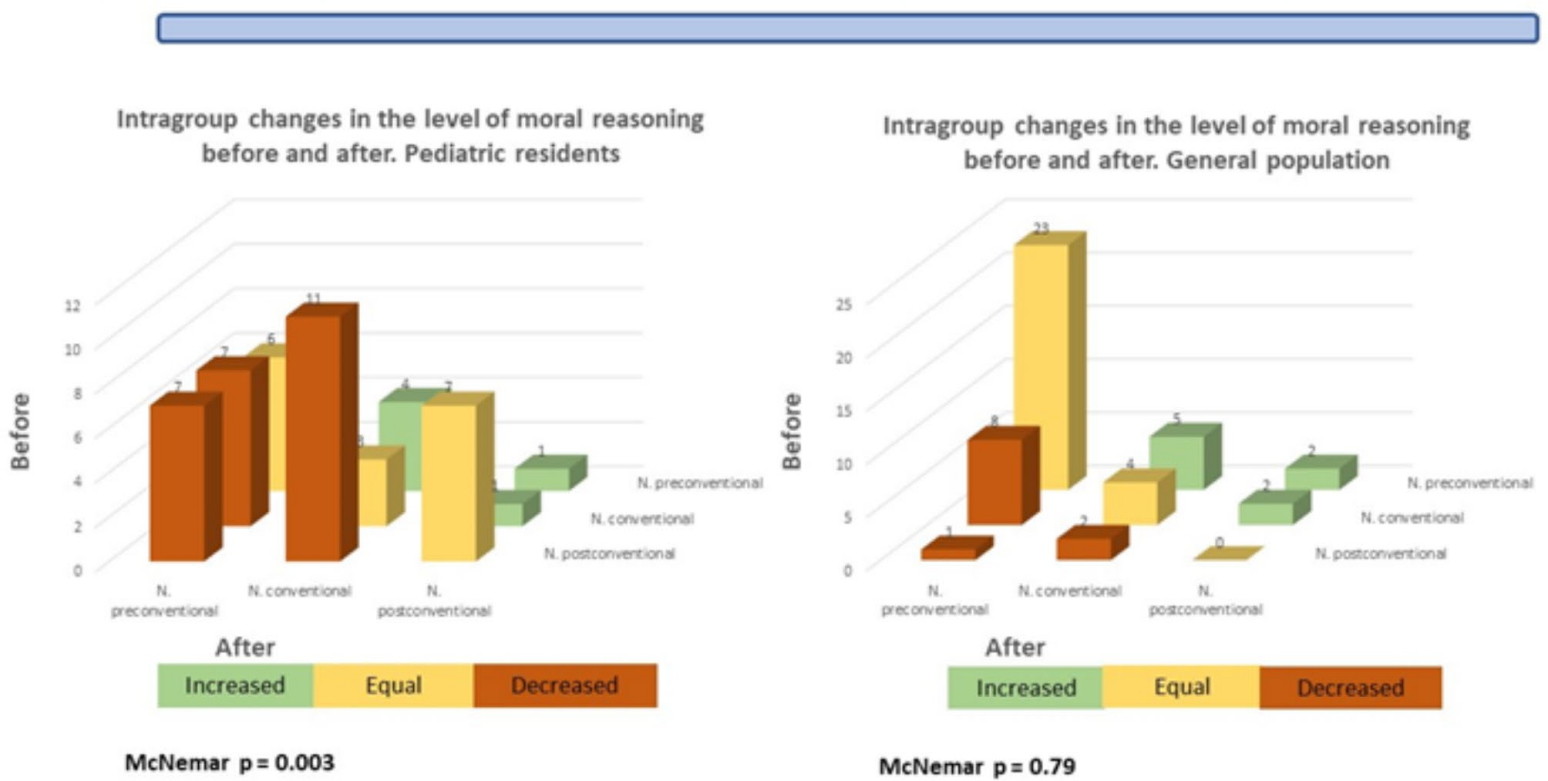
Changes in the level of moral reasoning before and after the pandemic

The changes in the stage of moral reasoning before and after the first year of the pandemic are shown in Fig. 2. In the group of residents, it was found that only 7 of 25 (28%) participants initially classified as postconventional remained at that stage; 11 (44%) fell to the conventional stage and 7 (28%) to the pre-conventional stage. As for the 11 participants who started at the preconventional stage, 6 (54%) remained at that stage, 4 (36%) increased to the conventional stage, and one to the postconventional stage. The McNemar-Bowker test was performed to rule out randomness, resulting in a p value of 0.003. In the general population group, it was found that the 3 participants who were initially at the postconventional stage decreased to stage: 2 went down to the conventional stage and one to the preconventional stage. As for the 30 participants who started at the preconventional stage, 23 remained at that stage, 5 went up to the conventional stage, and two to the postconventional stage, with no statistically significant differences being found.

## Discussion

This study is the first to assess changes in the moral development stage after a crisis such as the COVID-19 pandemic. We found crucial changes in the development of moral reasoning in pediatric residents after the first year of the pandemic compared to a general population group, where no such changes were found. Specifically, a decrease in the P index of 13 points (p = 0.002) was observed in pediatric residents compared to a decrease of 3 points (p = 0.4) in the general population group, which was not statistically significant. The decrease in stage observed among residents who were in the postconventional group is noticeable, with only 28% remaining at that stage after one year and up to 28% decreasing to the preconventional stage (p = 0.003). In the general population group, there was a decrease in the stage of 100% of the participants who were initially at the postconventional stage; however, this data is not statistically significant due to the small number of the sample (p = 0.79).

The decrease in P-score and developmental stages in medical students has been studied previously [30, 31]. Hren conducted a study with 707 third-year medical students and found a decrease from those who were initially within the postconventional stage to a conventional stage after one year of entering clinical practices, proposing that the hierarchical organization of clinical practices and the moral dilemmas that students might encounter could have a causal effect [32]. Meanwhile, Patenaude, in his study with 92 first-year medical students, found that after 3 years, 79% had decreased in stage. These findings contradict others that have not found changes over the years of studying medicine [33, 34]. Our population did not consist of undergraduate medical students but rather from the pediatrics specialty; however, it is possible that Hren’s findings about clinical practices and associated factors (hierarchical order and making moral decisions with patients) may partially influence the changes observed in our study.

In a previous study conducted by our research group (2019), which has not been published yet (under review) and which involves 88 pediatric residents from different years of the same hospital as the current study (first to third year of pediatrics and subspecialty students), a median P index of 33 was found (min-max range 3.3–76.6), with 37.5% of subjects in the postconventional stage, 26% in the conventional stage and 36% in the preconventional stage. These data differ from what was found in our study in the evaluation after one year of the pandemic, that is, when students entered the second year of the specialty, where we found a median P index of 30 (10–63.3), with only the 19% of subjects at the postconventional stage, 38% at the conventional stage and 43% at the preconventional stage. These important differences, especially in the percentage of subjects at the postconventional stage, allow us to know that not only clinical practices seem to be influencing, but also that the crisis due to the COVID-19 pandemic may be playing an important role.

It is known that an altered emotional state can modify the way people reason [23]. The COVID-19 pandemic has brought a psychological burden on the general population. In the case of health personnel, there is an abrupt transition from normal clinical practice due to the need to face a hitherto unknown disease, under extreme working conditions, with the constant fear of becoming sick or dying [35, 36]. So, the fact that the residents were on the front line of care for patients with COVID-19 could have a negative influence on their moral reasoning, which was not observed in the general population.

Conversely, the differences found in the baseline results between the two groups are considerable. Both groups showed a profile of moral development with a growth in the score from stage 2 to stage 4, with a predominance of the latter, to later descend in the higher stages. This same profile has been reported by other researchers [12–14]. However, the scores of the stages are different between the groups, with the high stages (5a, 5b, and 6) predominating in the group of residents and the low stages (2, 3, and 4) in the general population group. This is reflected in the differences found in the P index (PI), with 43 and 23 points for residents and the general population, respectively. These differences persisted despite the decrease observed in residents (PI 30 vs. 20 p = 0.01). This may be due to the educational stage of individuals, age, whether or not they profess a religion, or other circumstances.

It is important to take into account that the female sex was predominant in the total sample with 70%, because in both groups the sex distribution was the same, we do not consider that this influences the results, however, when performing a sub-analysis contemplating the entire population and dividing it by sex, we found that the baseline PI of women is slightly higher than that of men (PI 33.3 vs. 26.6, respectively) and the change observed after the first year of the COVID-19 pandemic was slightly greater in the group of women compared to men (6.7 points vs. 1.6 point PI decrease, respectively. This PI difference has also been observed in other studies [16, 37–39]. The study conducted by Self on 488 medical students found differences between the sexes of 5.5 to 8.4 points, among the 4 groups studied, favoring women [40].

Within the limitations of the study, the limited number of subjects per group and the differences in age and other sociodemographic variables between them should be considered, so the results are not fully generalizable, so it should be considered as an exploratory study that requires more evidence to make definitive conclusions. It is important to take into account that the educational level between the groups is different since all those in the group of pediatric residents have at least a bachelor’s degree, while in the general population group, all have a lower educational level, so it is not possible to you can rule out that this variable influences the results. The same with the monthly income, which tells us indirectly about the socioeconomic level of the participants, which can also influence the results. Therefore, we consider it important for future research to include a larger number of samples and thus be able to study the impact of these variables on moral reasoning and its stability or changes over time or in the face of life crises. Another limitation is that the population of residents was from a single specialty and institution, so the results may not be valid for other populations.

## Conclusions

After a year of the COVID-19 pandemic, we found a decrease in the stage of moral reasoning development in pediatric residents of a hospital converted to the care of patients with COVID-19, while it remained stable in the general population group. These changes may be explained by the hospital environment itself, with its hierarchical organization and day-to-day clinical decision-making, experienced by pediatric residents. However, being first-line physicians of patients with COVID may have influenced the changes found, which were not observed in the general population.

An important difference was found in the stages of moral reasoning between the group of pediatric residents compared to the general population group, finding a high prevalence of the preconventional stage in the general population and of the postconventional stage in pediatric residents. It is important for future research to consider a larger number of subjects to study the impact of age, economic and educational level on moral reasoning and its stability or change in the face of life crises.

## Electronic supplementary material

Below is the link to the electronic supplementary material.

Supplementary Material 1

## Data Availability

Data are available in Excel format on request. Point of Contact. Dr. Juan Garduño Espinosa. dilemasmorales20@gmail.com.

## Declarations

All methods were carried out in accordance with relevant guidelines and regulations.

All experimental protocols were approved by the Comité de Ética en Investigación del Hospital Infantil de México Federico Gómez, with register number CONBIOÉTICA-09-CEI-010-20160627.

## Abbreviations

DIT: Defining Issues Test
WHO: World Health Organization
