# Supplementary material for: How the COVID-19 pandemic affects the moral reasoning of pediatric residents and the general population
Source: BMC Med Educ. 2023 May 24;23:380. doi: 10.1186/s12909-023-04265-6 (PMC10208179; doi:10.1186/s12909-023-04265-6)
Supplement: Supplementary file 1 — Supplementary Material 1 [file 12909_2023_4265_MOESM1_ESM.docx]

**CRITERIA DEFINITION TEST**

**Instruction booklet**

The purpose of this questionnaire is to help us understand how people think about social problems. People have different opinions about what is right or wrong, fair or unfair. In the situations in this quiz, there are no 'correct' or exact answers in the way that math problems have exact answers. We want you to tell us what you think about various stories that present a problem.

You will be asked to first read a story in this notebook, and then record your answers on a special sheet.

To illustrate the procedure, an example is presented below:

**FRANCISCO AND THE CAR**

Francisco López is thinking of buying a car. He is married with two small children and earns a regular salary. The car you buy will be the only one for the family, it will be used mainly to go to work and transport the family in the city and, on some occasions, to go on vacation. When trying to decide which car to buy, Francisco realized that there are some things or issues that he has to take into account to judge its importance. For example: Should you buy a large but used car or a new but small car for about the same price? He has also thought about other questions.

We know that this is not a social problem, but it helps to exemplify the instructions. In the stories of social problems that this questionnaire contains you will express your opinions in three different sections, as explained below:

**SECTION ONE:** Express your opinion about what the person should do. If you prefer one action or another, indicate it even if you are not completely sure (a). If you don't agree with one, check the "can't decide" circle.

**SECOND SECTION:** read each of the statements numbered from 1 to 12 and reflect on the issue that the statement addresses. If you think that the issue is essential to make a decision in any sense, mark the option as very important. If you think the issue is not important or does not make sense to you, then mark no importance. If a statement is important, but not fundamental, mark a lot, some, or a little depending on how important you think it is. You can mark multiple statements with the same level of importance.

**THIRD SECTION:** after indicating the importance of each question, you are going to choose the most important statement of all based on the importance you already gave them before, even if you have not considered any of them to be very important. Then choose the second in order of importance, then the third, and finally the fourth. Be sure to note the importance you recorded in the second section.

Let's apply the above to Francisco's problem: it is about first recommending what he should do and then indicating the importance of the issues he should consider when making the decision. Observe how it proceeds following the three stages:

**SECTION ONE:** What should Francisco do?

-Buy a new car

-Buy a used car

-I can't decide.

**SECOND SECTION**: how important is each issue?

1. That the car shop is on the same block where Francisco lives

2. If a used car would be cheaper in the long run than a new one

3. That the car be green, which is Francisco's favorite color

4. That the cubic inch of displacement is at least 200

5. If a big, roomy car, it would be better than a small one

6. That the frontal thrusts are differential

**STATEMENTS**

| **IMPORTANCE** |  | **1** | **2** | **3** | **4** | **5** | **6** |
| --- | --- | --- | --- | --- | --- | --- | --- |
| Very much |  | O | O | O | O | O | O |
| A lot |  | O | O | O | O | O | O |
| Some |  | O | O | O | O | O | O |
| Little |  | O | O | O | O | O | O |
| None |  | O | O | O | O | O | O |

Note that the first statement was considered unimportant; the second was considered a fundamental matter, of great importance to make a decision; the third was considered to be moderately important or to have some importance; in the fourth, the respondent was not sure if 200 was good or not, so he marked "none"; the fifth statement was also judged to be of great importance and, finally, it was considered that the sixth did not make sense, for which reason "none" was also answered

**THIRD SECTION:** based on the answers of the previous section, the most important statement of all is chosen and its number is marked; then the second most important is chosen, and so on the third and fourth.

|  | **1** | **2** | **3** | **4** | **5** | **6** |
| --- | --- | --- | --- | --- | --- | --- |
| **Most important statement** | O | O | O | O | O | O |
| **Second most important statement** | O | O | O | O | O | O |
| **Third statement in importance** | O | O | O | O | O | O |
| **Fourth statement in importance.** | O | O | O | O | O | O |

Observe that when answering the third section the most important statement is one of those that were marked with great importance. To decide between statements 2 and 5, you must read them again and choose one of them as the most important and the other as the second in importance, until you point to the four most important.

Below is the first story that contains a social problem. Read it and then record your thoughts on each section on the answer sheet. After marking the four most important statements in the first story, go back to the text and read the second story, recording your responses in all three sections as in the first story. This is how he will attend the third story later.

Remember: when recording your answers be sure to fill in the ovals completely, keep the mark dark, and erase perfectly in case you need to make corrections. Use only the number 2.5 pencil (firm) to record your responses.

**_________________________________________________________________________**

**1st. STORY: ARMANDO AND MEDICINE**

In a Mexican city, a woman was about to die from a special type of cancer. There was a medicine that the doctors thought could save her. It was a medicine that a medicine manufacturer from the same city had recently discovered. The medicine was expensive to prepare, but the pharmacist charged ten times what it cost to make. He spent 2,000 pesos and charged 20,000 for a small amount of the medicine. The sick woman's husband, Armando, went with everyone he knew to get the money, but he could only raise 10,000 pesos; half the cost of medicine. He told the pharmacist that his wife was dying and asked him to sell it to him cheaper or to wait for it to pay him later, but the manufacturer said: "No, it took me a while to discover this medicine and I'm going to get all the money out of you." that I can".

Armando was about to get desperate and began to think about breaking into the man's store and stealing the medicine to give to his wife.

**SECTION ONE:** Should Armando steal the medicine?

**SECOND SECTION:** How important is each of the following questions?

1. The laws of the community are to be respected.

2. Isn't it natural for a husband who loves his wife to take such care of her to the point of stealing to help her?

3. Will Armando risk being shot as a robber or going to jail for the help he can get by stealing the medicine?

4. That Armando is a professional wrestler or has a lot of influence with professional wrestlers.

5. If Armando steals for himself or does it solely to help another person.

6. If the rights of the one who invented medicine have to be respected.

7. If the essence of living includes and surrounds the termination of dying, socially and individually.

8. What values ​​are the basis for regulating the way people act in their relationships with others?

9. Whether the pharmacist is going to be allowed to rely on an unfair law that only protects the rich anyway.

10. The law in this case attends to the most basic of rights of any member of society.

11. If the pharmacist deserves to be robbed for being so ambitious and cruel.

12. Will stealing in cases like this bring a greater good for all of society or not?

**THIRD SECTION:** Based on your opinions from the previous section, choose the four most important issues, ordering them from first to fourth.

**2nd. STORY: THE ESCAPED PRISONER**

One man was sentenced to prison with a ten-year sentence. After a year he ran away and went to live in a different part of the country and took the name, Martinez. For years he worked hard and saved little by little money to start his own business. During all this time he was fair to his customers, paid his employees very well, and gave most of his profits to charity. One day Mr. Pérez, a former neighbor, recognized him as the man who had escaped from prison eight years earlier and whom the police had been looking for. Mr. Pérez wondered if he should report Mr. Martínez to the police.

**SECTION ONE:** Should Mr. Pérez sue Mr. Martínez?

**SECOND SECTION:** How important is each of the following questions?

1. Hasn't Mr. Martinez been good enough long enough to prove he's not a bad person?

2. Every time someone escapes punishment for a crime, doesn't that encourage more crimes?

3. Wouldn't we be better off without prisons and the oppression of our legal system?

4. Has Mr. Martínez really paid his debt to the company?

5. Would the company be failing in what Mr. Martínez should reasonably expect of it?

6. What benefit prisons will be, except for society, especially for a charitable man?

7. How can someone be so cruel and heartless to send Mr. Martinez to prison?

8. Is it fair for the prisoners who are serving their full sentence that Mr. Martínez does not serve his?

9. Was Mr. Pérez a good friend of Mr. Martínez?

10. Isn't it the citizen's obligation to report a criminal who has escaped, regardless of the circumstances?

11. How could the will of the people and the common good be better served?

12. Going to prison, does Mr. Martínez do good or does he protect anyone?

**THIRD SECTION:** Based on your opinions from the previous section, choose the four most important issues, ordering them from first to fourth.

**3rd. STORY: THE SCHOOL NEWSPAPER**

Alfredo, a high school student, wanted to publish a newspaper made at his school so that he could express many of his opinions. He wanted to speak out against the use of weapons in international disputes and also against some school rules, like the one that forbids men from wearing long hair. To start his newspaper, Alfredo asked the management for permission. The director told him that was fine, as long as Alfredo promised to bring all the articles to him for approval before publication. Alfredo accepted and brought several articles to be approved. The director approved them all and Alfredo published two issues of the newspaper in the next two weeks.

However, the director did not expect Alfredo's newspaper to attract so much attention. The students were so enthusiastic about the paper that they began organizing protests against the ban on long hair and other school rules. Several parents became angry and opposed Alfredo's views; they telephoned the director and told him that the newspaper was unpatriotic and should not be published. As a result of growing discontent, the principal ordered Alfredo to stop publishing in the newspaper, telling him that the reason for suspending him was that his activities were interrupting the work of the school.

**SECTION ONE:** Should the editor suspend the newspaper?

**SECOND SECTION:** How important is each of the following questions?

1. Is the principal more accountable to students or to parents?

2. Did the editor give the newspaper a long-term license or promise to expressly approve each issue in due course?

3. Will the students start protesting even more if the principal suspends the newspaper?

4. When the welfare of the school is threatened, does the principal have the right to give orders to the students?

5. If there are conditions in the director's speech to say “no” in this case.

6. If the editor suspends the newspaper, will he be avoiding broad discussion of important issues?

7. The director's order will make Alfredo lose confidence in the director himself.

8. If Alfredo was really loyal to his school and patriotic in relation to his country.

9. What effect will the suspension of the newspaper have on the formation of judgment and critical thinking of students?

10. Whether Alfredo was in any way violating the rights of others by posting his own opinions.

11. If the principal should be swayed by some angry parents, he is the one who knows best what is going on at the school.

12. If Alfredo was using the newspaper to produce hatred and discontent.

**THIRD SECTION:** Based on your opinions from the previous stage, choose the four most important issues, ordering them from first to fourth.
